# Aortic Valve Fibroelastoma Masquerading as Transient Ischaemic Attack

**DOI:** 10.1155/2012/535783

**Published:** 2012-07-04

**Authors:** Penelope-Anne Gowland, Ragheb Hasan

**Affiliations:** Manchester Heart Centre, Manchester Royal Infirmary, Oxford Road, Manchester M13 9WL, UK

## Abstract

The following paper is on a 49-year-old man who presented to accident and emergency department having experienced five hours of left-sided chest pain, tightness in the chest, and shortness of breath. He also reported paresthesia and an ache in the left arm. Further investigations revealed an aortic valve papillary fibroelastoma. Although histologically papillary fibroelastomas are described as benign, they carry with them considerable risk of morbidity and mortality. This patient experienced recurrent transient ischemic attacks (TIAs'). He was taken to theatre on urgent basis to remove the papillary fibroelastoma. His aortic valve was preserved during the operation. The patient had an uneventful recovery following the surgery. His neurologic symptoms resolved following the operation. The operation was curative and no further symptoms were reported at followup.

## 1. Introduction

A 49-year-old man presented to accident and emergency department with five hours of left-sided chest painand shortness of breath. He was also complaining ofparesthesia and aching in the left arm. These symptoms were associated with nausea and general malaise.

His past medical history included hypothyroidism following thyroidectomy and hypertension. He works in engineeringand is a current smoker. His physical examination was unremarkable. Twelve-lead ECG showed high take off of the ST segment. Full blood count and chemistry profile were within normal range. Troponin T was not elevated (<0.01).

Two days following his admission, he complained again of paresthesia and heaviness in his left arm with mild motor dysfunction. Urgent brain CT was performed. This demonstrated multiple small foci (>10 mm) of low attenuation in the basal ganglia. These changes were attributed to periascular spaces (Virchow-Robin's spaces) rather than lacunar infarcts. No acute intracranial haemorrhagewas seen. No mass, midline shift or herniation was identified. Carotid Duplex scan was unremarkable. 

**Figure 1:**
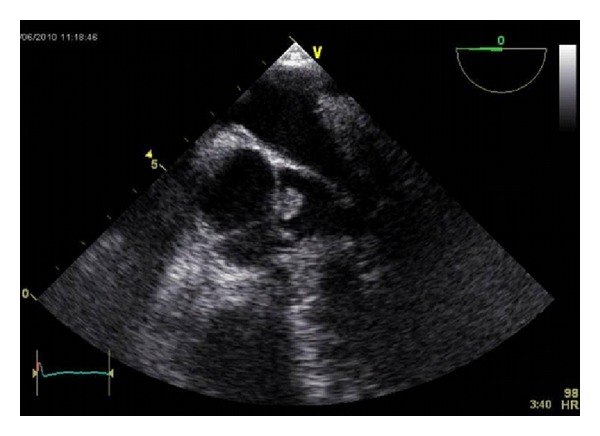
Echo image.

Echocardiogram showed a thin and mobile aortic valve with an echogenic mass attached to the noncoronary cusp of the aortic valve. This mass was located on the left ventricular side of the leaflet (Figure 1). The aortic sinuses were normal and no aortic regurgitation was detected. The left ventricular systolic function was uniformly good.

Although the patient has risks for coronary artery disease, it was felt that in the context of normal Troponin T and myocardial perfusion scan the potential risk of embolisation from the mobile mass on the aortic valve, coronary angiography was not indicated. Because of the recurrent transient ischemic attacks, it was prudent to offer our patient urgent operation to remove this mass.

**Figure 2:**
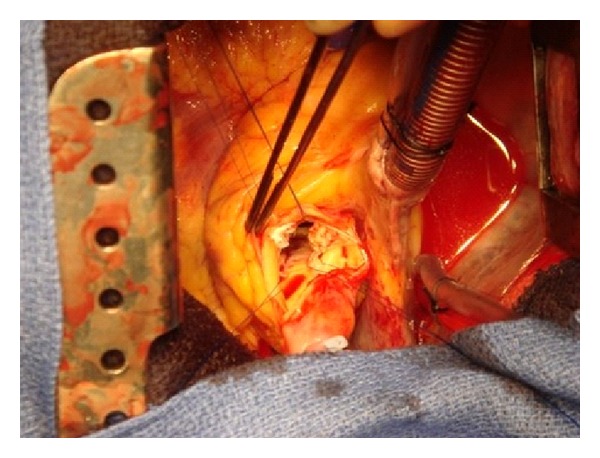
Perioperative view.

The patient was taken to theatre. Under cardiopulmonary bypass and moderate hypothermia, the aorta was cross-clamped and cold cardioplegia was infused down the aortic root. The heart arrested easily. The aorta was opened using a lazy S incision. On inspecting the aortic valve, there was a 0.5 inch mass on the ventricular side of the non coronary cusp (Figure 2). It looked like a fibroelastoma. The fibroelastoma was excised and the surrounding valvular endothelium on the ventricular side of the cusp but preserving the fibrosa and the endothelium on the aortic side. The valve was competent. Rewarming was commenced. The aortotomy was closed in double layer fashion using continuous 5/0 prolene sutures. The cross-clamp was removed and the heart resumed sinus rhythm. The patient was weaned off cardiopulmonary bypass uneventfully. Postoperative transoesophageal echo confirmed normal aortic valve function and no aortic regurgitation.

**Figure 3:**
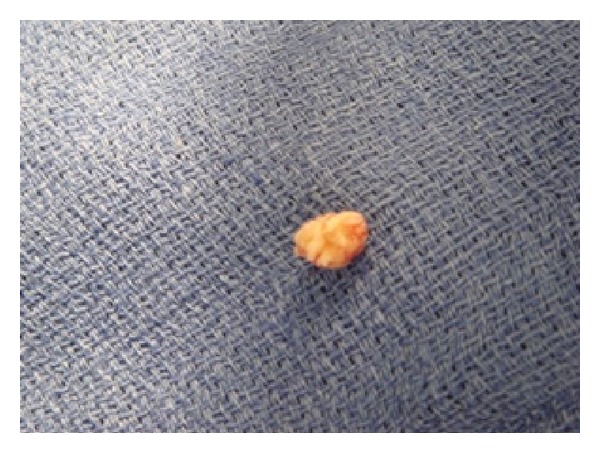
Histology specimen.

Histopathology report confirmed a pale grey piece of tissue measuring 0.8 × 0.7 × 0.5 cm. Microscopic sections revealed a collagenous/hyalinised tissue with areas of myxoid change. There was short avascular papillary branching lined by endothelium. The features were consistent with the clinical suspicion of fibroelastoma (Figure 3). 

The patient had an uneventful postoperative course and was discharged home on the fifth postoperative day. The patient was seen at the outpatient clinic. All his symptoms had resolved. Follow-up echocardiogram demonstrated normal aortic valve and left ventricular function.

## 2. Discussion

Although histologically papillary fibroelastomas are described as benign, they carry with them considerable risk of mortality and morbidity.They cause peripheral emboli with end organ damage, arrhythmias, and acute valvular dysfunction. The embolic and mechanical consequences arising from the papillary fibroelastoma can cause permanent dysfunction or catastrophic events including sudden death [1, 2].

It was intriguing why this man experienced so many symptoms although the papillary fibroelastoma was small and on the ventricular side of the noncoronary cusp of the aortic valve.Literature search did not reveal any publications that evaluated the dynamics of blood flow in the noncoronary cusp and sinus. There are rare reports of thromboembolism arising from the non coronary cusp. Shindo et al. [3] hypothesize that blood flow in the non coronary cusp is different from the two coronary cusps and that stagnation and vortices of blood flow during diastole could potentially be contributing factors for thrombotic events. 

The incidence of cardiac tumours is thought to be approximately 0.02% [4]. The most common presenting symptom is cerebral embolism [5]. Asymptomatic cases of papillary fibroelastoma are increasingly being diagnosed as cardiac imaging becomes more widely used. The indications for considering surgery are patient presenting with symptoms and/or a mobile tumour [4]. Surgical removal of the papillary fibroelastoma with preservation of the valve is a curative [5–7]. In our case, we excised the tumour with total preservation of the aortic valve.

## 3. Conclusion

We report a patient who presented with angina-like chest pains and TIAs. The patient was subsequently found to have a papillary fibroelastoma. He underwent successful aortic valve sparing resection of the tumour and had an uneventful post operative course. The uniqueness about this case was the severity of symptoms the patient experienced from such a small cardiac tumour on the non coronary cusp. His prompt diagnosis and treatment protected him from any permanent disability. The patient kept his native valve.

At post-operative followup, he had complete resolution of his symptoms and a structurally normal aortic valve with no regurgitation.
